# Successful bypass operation for esophageal obstruction after acute esophageal necrosis: a case report

**DOI:** 10.1186/s40792-016-0277-8

**Published:** 2017-01-04

**Authors:** Yayoi Sakatoku, Masahide Fukaya, Kazushi Miyata, Masato Nagino

**Affiliations:** Division of Surgical Oncology, Department of Surgery, Nagoya University Graduate School of Medicine, 65 Tsurumai-cho, Showa-ku, Nagoya, 466-8550 Japan

**Keywords:** Esophageal bypass, Acute esophageal necrosis, Esophageal stricture

## Abstract

**Background:**

Acute esophageal necrosis (AEN) is a rare clinical disorder. Esophageal stenosis or obstruction is one of severe complications, but there are a few reports about surgical treatments. In such a situation, it still remains controversial which to choose, esophagectomy or bypass operation.

**Case presentation:**

A 61-year-old woman was admitted to the local hospital for septic shock with diabetic ketoacidosis due to necrotizing fasciitis of the right thigh. Three days later, she had hematemesis, and gastrointestinal endoscopy revealed black mucosal coloration throughout the entire esophagus. She was diagnosed as having AEN. Her general condition improved after intensive care, debridement, and treatment with antibiotics and a proton pump inhibitor; the esophageal mucosal color recovered. However, an esophageal stricture developed after 1 month, and the patient underwent gastrostomy to remove an esophageal obstruction after 3 months. She was referred to our hospital for surgical treatment 1 year and 4 months after the occurrence of AEN because of her strong desire for oral intake. Her medical condition was poor, and she could not walk due to generalized muscle weakness. After rehabilitation for 8 months, we performed an esophageal bypass using a gastric conduit via the percutaneous route rather than esophagectomy because of her multiple severe comorbidities including walking difficulty, chronic hepatitis C, cerebrovascular disease, and chronic renal failure. Minor leakage of the esophagogastrostomy occurred and was resolved with conservative treatment. The patient began oral intake on postoperative day 34 and was discharged on day 52.

**Conclusion:**

Esophageal obstruction after AEN was successfully treated by esophageal bypass using a gastric conduit in a high-risk patient. Because the majority of patients with AEN have multiple severe comorbidities, assessing the medical condition of the patient adequately is important prior to choosing either an esophagectomy or bypass surgery.

## Background

Acute esophageal necrosis (AEN), or black esophagus, is endoscopically defined as diffuse dark pigmentation of the esophageal wall and is a rare clinical disorder. The etiology of AEN remains unclear and is likely multifactorial. Gurvits et al. [1] reviewed 88 cases of AEN and reported that the risk factors included old age, male sex, cardiovascular disease, hemodynamic compromise, gastric outlet obstruction, alcohol ingestion, malnutrition, diabetes, renal insufficiency, hypoxemia, hypercoagulable state, and trauma. Complications related to AEN included strictures (10.2%), mediastinitis/abscess (5.7%), and perforation (6.8%), and the overall mortality rate was 31.8% [1].

The treatment of an esophageal stricture due to AEN is comparable to the treatment of peptic esophageal stricture, which is treated with bougies or endoscopic balloon dilation (EBD), steroid injections, and surgery [2]. Although esophageal stricture after AEN is often refractory to conservative therapy, there are a few reports of surgical intervention [3–5]. Herein, we report a rare case of successful esophageal bypass for esophageal obstruction after AEN.

## Case presentation

**Fig. 1:**
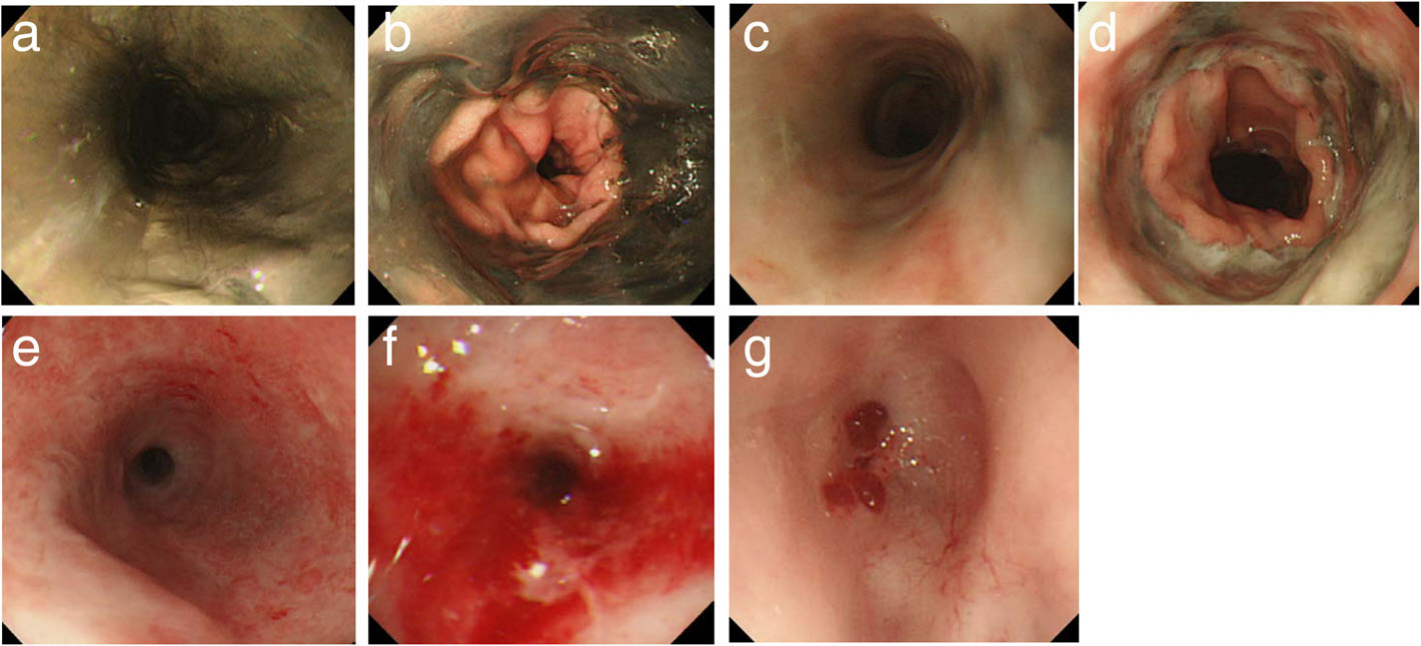
Acute esophageal necrosis (AEN). An emergent gastrointestinal endoscopy revealed a so-called black esophagus with color change of the mucosa throughout the entire esophagus (**a**). The lesion abruptly stopped at the gastroesophageal junction (**b**). Endoscopy on day 10 after the onset of AEN revealed that the color of the mucosa was partially improved (**c**, **d**). Esophageal stricture began approximately 1 month after AEN (**e**). Three months later, endoscopy revealed a pinhole stenosis of the upper thoracic esophagus (**f**). Two years after AEN, endoscopy revealed the esophageal obstruction with no findings of malignancy (**g**)

A 61-year-old woman with poorly controlled diabetes mellitus and malnutrition was admitted to the local hospital for necrotizing fasciitis of the right thigh. She developed septic shock with diabetic ketoacidosis. Three days later, she had hematemesis, and gastrointestinal endoscopy revealed circular black mucosal changes throughout the entire esophagus (Fig. 1a, b). She was diagnosed as having AEN. As her general condition improved with intensive care, debridement, and treatment with antibiotics and a proton pump inhibitor, the color of the mucosa partially improved (Fig. 1c, d). One month later, endoscopy showed an esophageal stricture of the upper thoracic esophagus (Fig. 1e), which progressed to an esophageal obstruction after 3 months (Fig. 1f). She underwent open gastrostomy due to her inability to eat. She was referred to our hospital for surgical treatment 1 year and 4 months after the occurrence of AEN because of her strong desire for oral intake.

**Fig. 2:**
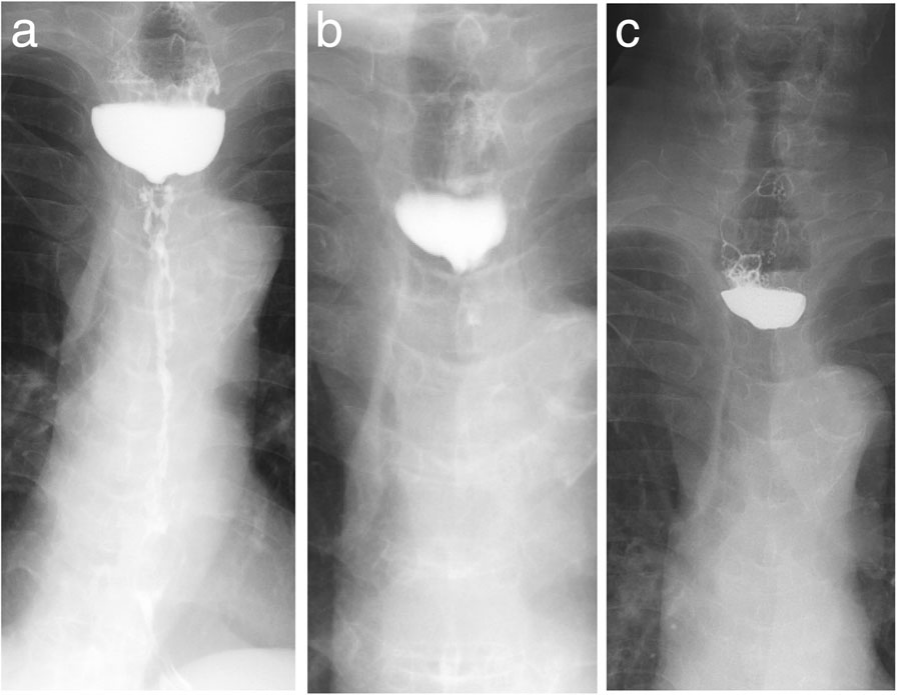
Two months after AEN, the upper gastrointestinal image (UGI) confirmed a diffuse esophageal stricture from the upper to the lower esophagus (**a**). Three months later, the UGI confirmed that the Gastrografin stopped the stricture at the upper border of the clavicle (**b**). Two years after AEN, the UGI confirmed complete esophageal obstruction (**c**)

**Fig. 3:**
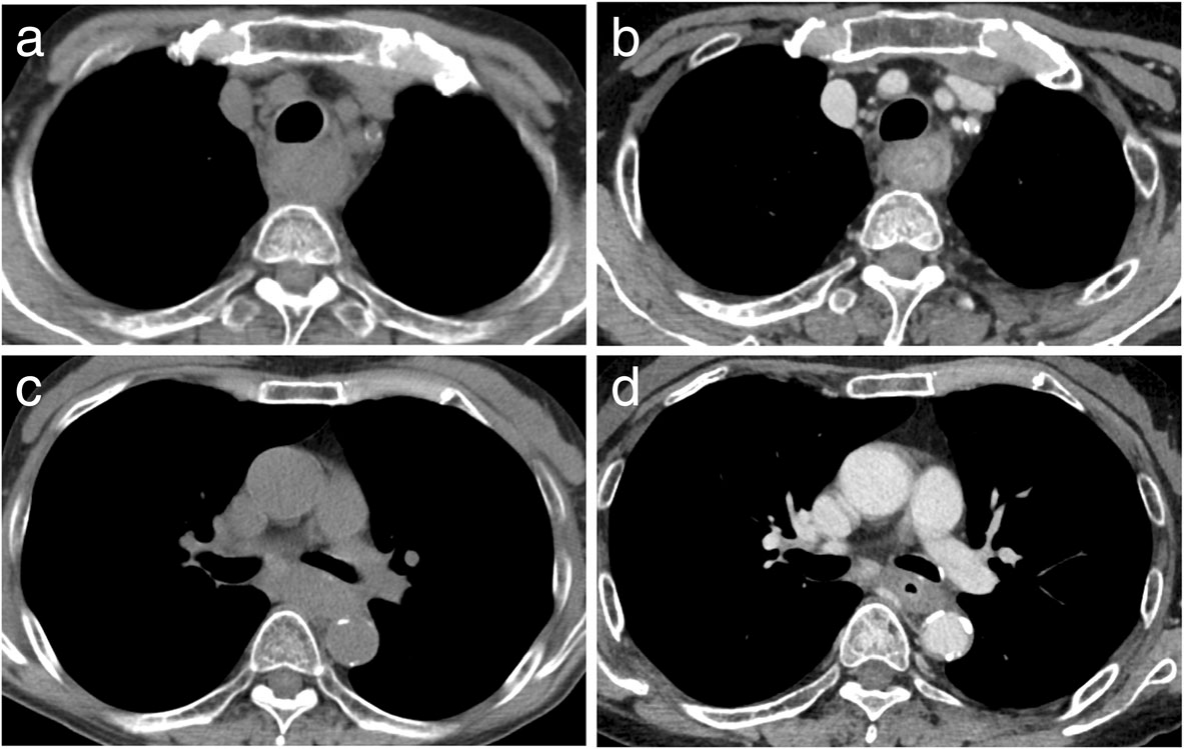
Computed tomography (CT) after AEN showed circumferential wall thickening of the upper to the lower thoracic esophagus: upper esophagus (**a**) and middle esophagus (**b**). Two years after AEN, a CT scan showed similar findings: upper thoracic esophagus (**c**) and middle thoracic esophagus (**d**)

Endoscopy revealed an esophageal obstruction, and the biopsied specimen at the blind end of the esophagus revealed esophagitis without any malignancy (Fig. 1g). Upper gastrointestinal imaging (UGI) confirmed complete esophageal obstruction at the upper border of the clavicle (Fig. 2). Computed tomography (CT) showed circumferential wall thickening from the upper to middle thoracic esophagus (Fig. 3). When she was referred to our hospital, her medical condition was poor, and she could not walk due to generalized muscle weakness. After rehabilitation for 8 months, she underwent esophageal bypass.

**Fig. 4:**
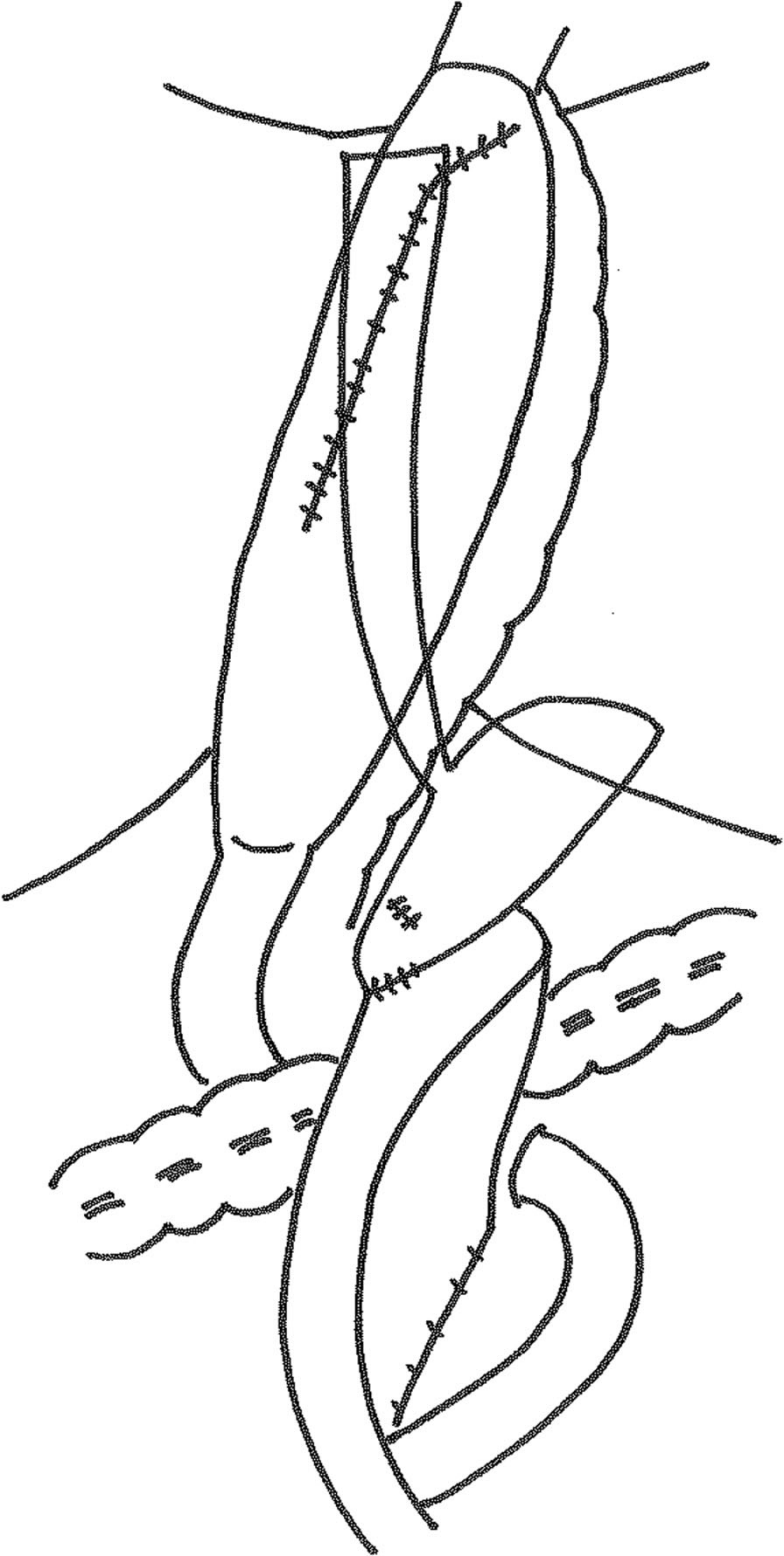
The schema of the reconstruction. We performed an esophageal bypass using a gastric conduit via the percutaneous route. A gastric tube, excluding the insertion site of the gastrostomy, was created. The remnant stomach with the left gastric vessels was retained, and we performed a gastrojejunal anastomosis via an antecolic Roux-en-Y reconstruction

First, the cervical esophagus was cut at the level of the suprasternal notch. In the initial plan, pedunculated jejunum was to be used for the esophageal reconstruction because of the uselessness of the stomach due to the gastrostomy. However, the shortening of the small bowel mesentery would not allow for a reconstruction with pedunculated jejunum. Fortunately, a gastric tube, excluding the insertion site of the gastrostomy, could be created, and the esophageal bypass was performed using a gastric conduit via the percutaneous route. The remnant stomach with the left gastric vessels was preserved, and a gastrojejunal anastomosis via an antecolic Roux-en-Y reconstruction was performed (Fig. 4).

During the postoperative course, minor leakage of the esophagogastrostomy occurred on day 6 and was resolved with conservative treatment. The patient began oral intake on postoperative day 34 and was discharged on day 52. One year after surgery, the patient had good oral intake with good nutrition, although hemodialysis was initiated due to worsening of her chronic renal failure.

### Discussion

The management of AEN is divided into acute and chronic phases. In the acute phase, intensive care for underlying diseases is important and sequential life-threatening complications, such as esophageal perforation, mediastinitis, and abscess, require emergent esophagectomy or drainage. In the chronic phase, esophageal stricture requires EBD, esophagectomy, or a bypass operation. In the present case, the patient’s general condition improved with intensive care, debridement, and antibiotics for necrotizing fasciitis of the right thigh, and esophageal perforation did not occur during the acute phase. However, the diffuse stricture extended over the entire esophagus, and an esophageal obstruction developed after 3 months.

A review of the literature found that 19 of 163 patients with AEN progressed to esophageal stricture 3 weeks to 2 months after the onset of AEN [3, 6–13]. Gurvits et al. reported that coronary artery disease, recent surgery, and gastroduodenal pathology may be associated with esophageal stricture [1]. Shichinohe et al. [3] reported three cases of acquired esophageal stricture after AEN with septic shock and hypothesized that severe hypoperfusion of the middle to low esophagus due to septic shock may cause esophageal stricture after AEN. Our patient developed a state of severe septic shock and had both a hiatal hernia and duodenal ulcer at the time of the AEN diagnosis. The extent and duration of septic shock and the extent of acid reflux to the esophagus may be associated with esophageal stricture after AEN.

Based on a review of the literature, the estimated treatment prevalence for post-AEN esophageal stricture is very low. The majority of the 19 patients with esophageal stricture initially underwent EBD. However, five patients received surgical treatment for refractory esophageal stricture after repeated EBD, three patients underwent esophagectomy, and two patients had a bypass operation [3–5]. In most patients without surgical treatment, the follow-up duration was only a few months; therefore, more patients may have received surgical treatment. Our patient never received EBD but underwent open gastrostomy due to esophageal obstruction after 3 months. EBD should have been considered before the esophageal stricture progressed to obstruction. Though an esophageal stent have been reported to be useful for refractory benign esophageal stricture [14, 15], there is a report that an esophageal stent placement itself can be a risk factor related to AEN [16], and no report about successful stent placement for esophageal stricture after AEN. Therefore, stent treatment for esophageal stricture after AEN still remains controversial. Furthermore, we consider an esophageal stent is not suitable for this patient because placement of a stent in the cervical and upper thoracic esophagus can cause severe discomfort.

With respect to the requirement for esophagectomy, corrosive esophageal stricture and esophageal achalasia have been reported to increase the risk of carcinogenesis due to chronic inflammation [17], and esophagectomy rather than bypass surgery is the preferred procedure. In our case, a transthoracic or transhiatal esophagectomy was considered to be difficult for the patient to receive because of her poor medical condition, including walking difficulty, chronic hepatitis C, cerebrovascular disease, and chronic renal failure due to diabetic nephropathy (serum creatinine levels 2.3 mg/dl). We found no clinical evidence for malignancy in the esophagus, and sequential CT images after AEN showed no changes. However, there was circumferential wall thickening from the upper to the middle thoracic esophagus; therefore, we chose a bypass operation rather than esophagectomy.

Regarding the reconstructive method, the pedunculated jejunum with microvascular anastomosis was proposed because of the uselessness of the stomach due to a gastrostomy; however, the length of the pedicle of the small intestine was inadequate to anastomose to the cervical esophagus. For the same reason, a Y-shaped gastric tube for reconstruction was not used. We were able to perform an esophageal bypass using a gastric conduit, excluding the insertion site of the gastrostomy. An interruption in the circulation around the esophagogastric junction was considered because endoscopy at the onset of AEN revealed the presence of black mucosal discoloration; consequently, we did not cut the esophagogastric junction or perform esophagojejunostomy. Instead, we retained the remnant stomach and performed a gastrojejunostomy to avoid anastomotic leakage of the esophagojejunostomy.

## Conclusions

Esophageal obstruction after AEN was successfully treated by esophageal bypass using a gastric conduit in a high-risk patient. Because the majority of patients with AEN have multiple severe comorbidities, assessing the medical condition of the patient adequately is important prior to choosing either an esophagectomy or bypass surgery.

## Abbreviations

AEN: Acute esophageal necrosis
CT: Computed tomography
EBD: Endoscopic balloon dilation
UGI: Upper gastrointestinal image
